# Magnetoliposomes with size controllable insertion of magnetic nanoparticles for efficient targeting of cancer cells†

**DOI:** 10.1039/c9ra02529d

**Published:** 2019-05-14

**Authors:** Won Il Choi, Abhishek Sahu, Frederik R. Wurm, Seong-Min Jo

**Affiliations:** Center for Convergence Bioceramic Materials, Convergence R&D Division, Korea Institute of Ceramic Engineering and Technology 202, Osongsaengmyeong 1-ro, Osong-eup, Heungdeok-gu Cheongju Chungbuk 28160 Republic of Korea; School of Materials Science and Engineering, Gwangju Institute of Science and Technology 123 Cheomdan Gwagi-ro, Oryong dong, Buk-gu Gwangju 61005 Republic of Korea; Max Planck Institute for Polymer Research Ackermannweg 10 D-55128 Mainz Germany jos@mpip-mainz.mpg.de seongmini@gmail.com

## Abstract

Liposomes with embedded magnetic nanoparticles (magnetoliposomes; MLs) are promising nano-platforms for various biomedical applications. The magnetic behavior of MLs depends on the size of embedded magnetic nanoparticles (MNPs); in general, larger MNPs are more advantageous (*e.g.* increased magnetic signals). However, the insertion of large MNPs into liposome bilayers is constrained by the thickness of the membrane (∼3.4 nm); thus, the incorporation of larger magnetic nanoparticles (>3.4 nm) into liposomes is a major challenge. We developed a solvent-guided approach for the simple and efficient insertion of large MNPs (6 nm or 15 nm) into the liposomal bilayer. MLs with 6 nm MNPs were used for the magnetic field-guided separation of cancer cells by targeting to human epidermal receptor 2 and folate receptor. We also evaluated the nuclear delivery of oligonucleotides by MLs with a cationic lipid formula. The MLs are expected to be versatile nano-platforms for biomedical applications (*e.g.* disease diagnosis, therapeutics and cell tracking).

## Abstract 2

Cationic magnetoliposomes enable to achieve efficient capture of cells and intracellular delivery of oligonucleotides into nucleus through cancer cell-specific targeting.

## Graphical abstract

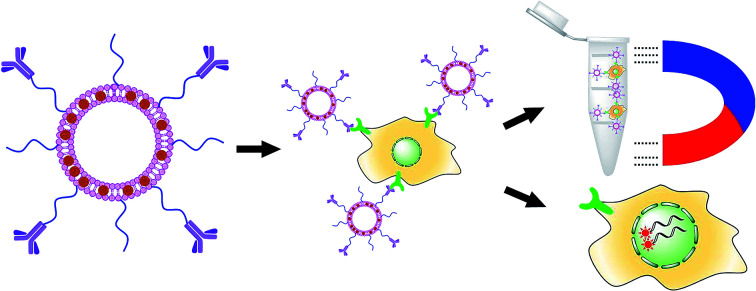

## 1. Introduction

Combinations of functional inorganic nanoparticles with liposomes have various applications in biomedical research.^1–3^ Among these organic–inorganic hybrid nanoassemblies, magnetic nanoparticle-embedded liposomes, commonly referred as magnetoliposomes (MLs), are a promising multifunctional platform for bio-imaging, drug delivery and control the cell signaling.^4,5^ MLs are frequently used as contrast agents for magnetic resonance imaging (MRI) and as carriers for chemotherapeutic drugs.^6–8^ Additionally, MLs are useful for magnetic field-guided targeted drug delivery, on-demand controlled drug release, hyperthermia therapy, cell tracking, cell sorting, and intracellular transportation, among other applications.^9–13^ The size of magnetic nanoparticles (MNPs) is a key determinant of their magnetic properties.^14,15^ The magnetization and T_2_ relaxation of MNPs generally increases as the particle size increases.^16,17^ Jun *et al.* demonstrated that the magnetization and T_2_ relativity of Fe_3_O_4_ nanocrystals increased by ∼4 fold by increasing the particle size from 4 to 12 nm.^18^ The 12 nm Fe_3_O_4_ nanocrystals exhibited better contrast enhancement in MR imaging and improved tumor detection than those of 4 nm nanocrystals.^18^ Similarly, MLs with stronger magnetic properties could be more useful for biomedical applications. As the magnetic properties of MLs are solely dependent on the encapsulated MNPs, the incorporation of larger MNPs to liposomes with high efficiency is of great interest.

Conventional methods for the insertion of inorganic/metal nanoparticles into liposomes are based on hydrophobic interactions between the aliphatic hydrocarbons of the phospholipid bilayers and the lipid surface of the nanoparticles.^19–21^ Nanoparticles with hydrophobic surface coating are spontaneously embedded into the phospholipid bilayers of liposomes during the hydration of the lipid film. However, the size of nanoparticles that can be embedded into liposomes by the conventional approach is limited by the thickness of the membrane. In general, the phospholipid bilayer structure of the liposome is approximately 3.4 nm thick, which makes the insertion of any nanoparticle exceeding that size a challenging task.^22,23^ Chen and co-workers proposed a ‘neighboring membrane model’ to insert superparamagnetic iron oxide (SPIO) nanoparticles of approximately 5 nm in diameter into the bilayers of liposomes prepared from dipalmitoyl phosphatidylcholine (DPPC).^24^ Even though the model is reasonable, this method has a low nanoparticle insertion efficacy. In another approach, Bonnaud *et al.* reported that the addition of a surfactant enables the insertion of larger MNPs (6.5 nm in diameter) into the liposomal membrane with high efficiency.^25^ However, small molecular surfactants are difficult to completely remove from the final product and can have negative effects on the stability of nanoparticle-embedded liposomes.

Here, we demonstrate a solvent-guided method for efficient preparing MLs with large MNPs (diameters of ∼6 nm and ∼15 nm) inserted into the phospholipid bilayers (∼3.4 nm in thickness). Typically, nanoparticles with average diameters exceeding the bilayer thickness are only sparingly allowed into phospholipid bilayers.^26^ According to our preliminary results, ∼6 nm MNPs were difficult to embed into phospholipid bilayers by a conventional dried-film hydration method. To enhance the insertion efficiency, we used chloroform as a supporting agent to guide the MNPs into the phospholipid membrane.

## 2. Experimental section

### 2.1. Materials

1,2-Dioleoyl-*sn*-glycero-3-phosphocholine (DOPC), 1,2-dioleoyl-3-trimethylammonium-propane (DOTAP), distearoylphosphatidylethanolamine (DSPE)-mPEG_2000_, and DSPE-PEG_2000_-folate were purchased from Avanti Polar Lipid (Alabaster, AL, USA). l-α-Phosphatidylcholine from egg yolk (Egg PC) and HEPES were obtained from Sigma-Aldrich (St. Louis, MO, USA). Magnetic nanoparticles (15 nm) were purchased from Ocean Nanotech (San Diego, California, USA). NHS-PEG_3400_-maleimide was obtained from Laysan Bio (Arab, AL, USA). *N*-Succinimidyl *S*-acetylthioacetate was purchased from Thermo Scientific (Waltham, MA, USA). The anti-HER2 antibody was purchased from Roche (Basel, Switzerland). All other chemicals and solvents were purchased from Sigma-Aldrich and were of ACS reagent grade or biotechnology grade.

### 2.2. Cell culture

SK-Br3 and HeLa cells were obtained from the ATCC (American Type Culture Collection, Manassas, VA, USA). Both cancer cells were cultured in RPMI1640 media supplemented with 10% FBS in a CO_2_ incubator (37 °C, 5% CO_2_). Dulbecco's buffered saline (DPBS) was used for all cell washing steps. For cell detachment, trypsin/EDTA solution was used. All reagents for cell culture were purchased from Thermo Scientific (Waltham, Massachusetts, USA).

### 2.3. Preparation of MLs

The 6 nm MNPs were synthesized according to previously described methods.^27^ To prepare the MLs, 10 mg of the phospholipid mixture (DOPC : DOTAP = 7 : 3 molar ratio) and 0.5 mg of magnetic nanoparticles were mixed in 1 mL of chloroform. In a glass flask, a thin lipid layer was generated by evaporating the solvent and the lipid film was hydrated by adding 1 mL of HEPES buffer (150 mM, pH 7.4) and 0.1 mL of chloroform. Emulsification was performed using a sonication bath for 10 min and then chloroform was evaporated by stirring the open vessel at 45 °C. For the PEGylation of MLs, a 2% molar ratio of DSPE-mPEG_2000_ was further added to the lipid mixture during lipid film preparation.

### 2.4. Antibody thiolation

The anti-HER2 antibody (0.5 mg) was reacted with an 18 molar excess of succinimidyl acetylthioacetate for 45 min. After the reaction, 0.05 mL of hydroxylamine hydrochloride solution (0.5 M) was added to the reaction mixture and further incubated for 2 h. The thiolated antibody was purified by removing byproducts and unreacted reagents using a dextran desalting column. The concentration of the antibody was determined by absorbance measurement.

### 2.5. Preparation of antibody-conjugated MLs

MLs (5 mg) were reacted with 1.36 mg of NHS-PEG_3400_-maleimide for 45 min. The maleimide-functionalized MLs were purified using a centrifugation filter (100k MWCO) and 0.5 mg of thiolated anti-HER2 antibody was added to this ML suspension. After 4 h of reaction at room temperature, the resulting antibody-conjugated MLs were purified using a centrifugation filter (300k MWCO). The amount of antibody bound to the MLs was quantified by measuring the protein concentration in the supernatant. The antibody (0.12 mg) was conjugated to 5 mg of MLs. To load the oligonucleotides, 100 pmol Atto590-labeled 18 nt oligo DNA (AGC TGC TCT AGT ATC TGC; 5466 daltons) was mixed with 100 μg of MLs. After incubation for 30 min, the unbound DNA was removed using a centrifugal filter (300k MWCO).

### 2.6. Preparation of folate-modified MLs

Briefly, 10 mg of phospholipid (DOPC : DOTAP = 7 : 3 molar ratio), 0.5 mg of magnetic nanoparticles, DSPE-PEG_2000_-folate (0.01 molar amount against total lipids), and DSPE-mPEG_2000_ (0.025 molar amount against total lipids) were mixed in chloroform and a thin lipid layer was generated on the wall of the glass flask. Then, 1 mL of HEPES buffer (150 mM, pH 7.4) and 0.1 mL of chloroform were added to the dried film for hydration. Emulsification was performed using a bath-type sonicator for 10 min, and chloroform was evaporated.

### 2.7. Cancer cell isolation test

The suspension of SK-BR3 or HeLa cells containing 5.0 × 10^4^ cells per mL was prepared in serum-free RPMI1640 media. For folate-targeting experiments, folate and serum-free MEM was used. The ML suspension of 50 μL (2 mg mL^−1^) was added to each cell suspension of 1 mL, and was gently mixed for 15 min. Then, the cells were magnetically isolated, and the cell pellet was washed twice with DPBS. The number of cells recovered was counted using a hemocytometer. The isolation efficiency was calculated by comparing the initial number of cells and the number of isolated cells.

### 2.8. Intracellular delivery of MLs

The cancer cells (SK-Br3) were cultured on an 8-channel glass chamber slide. The attached cells were treated with 200 μL of the Atto590-labeled 18 nt oligo DNA loaded ML suspension (10 μg mL^−1^). After 20 min of incubation, the MLs were washed with DPBS and fresh serum-free media was added to the wells. After further incubation for 3 h in typical cell culture conditions (37 °C, 5% CO_2_), cells were fixed with 4% paraformaldehyde and the slides were observed under a confocal microscope.

## 3. Results and discussion

### 3.1. Attempts to prepare MLs by spontaneous insertion

**Fig. 1:**
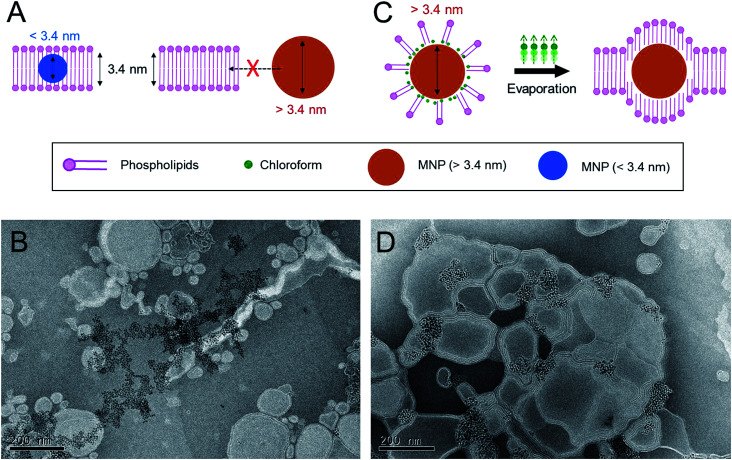
Proof-of-concept for chloroform-guided insertion. (A) Schematics of insertion of the nanoparticles into phospholipid membrane. Smaller nanoparticles below thickness of the membrane (3.4 nm) could be embedded, but larger nanoparticles could not enter to the membrane. (B) Trying to prepare the MLs by sonication methods without chloroform-guidance. MNPs (6 nm) could not be inserted. (C) Chloroform-guided method enables insertion of the larger nanoparticles into the membrane. (D) Preparation of MLs by sonication and chloroform-guided methods. MNPs (6 nm) could be placed in phospholipid membranes.

First, we tried to evaluate the insertion of moderately sized MNPs with diameters of around ∼6 nm into the phospholipid bilayers of cationic liposomes by the conventional dried-film hydration method, which relies on spontaneous hydrophobic interactions (Fig. 1A). For this purpose, the MNPs consisted of iron oxides as core materials and oleic acid as a surface-coating material, prepared by a thermal decomposition method described previously.^27^ A lipid formulation of a 7 : 3 molar mixture of 1,2-dioleoyl-*sn*-glycero-3-phosphocholine (DOPC) to 1,2-dioleoyl-3-trimethylammonium-propane (DOTAP), which is widely used for intracellular delivery, was used to prepare the MLs in this study.^28–30^ The estimated thickness of the phospholipid bilayers of the liposomes prepared from this lipid composition is ∼3.4 nm.^22^ Previous studies have demonstrated that MNPs with an average size of less than 3.4 nm can be readily incorporated into the phospholipid bilayer of this type of cationic liposome using a conventional film rehydration method.^21,31^ To prepare MLs by the conventional approach, DOPC, DOTAP, and MNPs were mixed in an organic solvent, and the solvent was then completely removed under vacuum. The dried lipid film was hydrated in HEPES buffer solution (150 mM and pH 7.4), producing a minimally dispersed aqueous phase. The incomplete mixture was further sonicated. However, a large amount of brown precipitate was observed, implying that most of the MNPs were not incorporated into the liposome bilayer and consequently aggregated to form a precipitate in the aqueous phase. Transmission electron microscopy (TEM) (Fig. 1B) clearly showed that most of the MNPs aggregated outside of the liposomes; it was extremely difficult to find well-formed MLs. This result suggests that MNPs with diameters of ∼6 nm are unfavorable for spontaneously entering the ∼3.4 nm-thick phospholipid bilayer solely *via* hydrophobic interactions. Based on this preliminary result, we concluded that the conventional method is not sufficient to efficiently incorporate MNPs with a diameter exceeding the thickness of the bilayer membrane of phospholipid liposomes. Therefore, a different method is needed to achieve this objective.

### 3.2. Preparation of MLs with 6 nm MNPs by solvent-guided methods

We hypothesized that the application of additional drag force from the aqueous phase to lipid bilayers would enhance the insertion efficiency of MNPs into the liposome bilayer. To determine the effective drag force, we focused on the chemical properties of chloroform, an organic solvent routinely used to solubilize lipids (Fig. 1C). Chloroform is immiscible with an aqueous phase but is a good solvent for both phospholipids and lipid-coated nanoparticles. Accordingly, we fabricated MLs with chloroform as a supporting agent. DOPC, DOTAP, and ∼6 nm MNPs were dissolved in chloroform, but instead of evaporating the chloroform to form a lipid film, we emulsified the organic phase directly into the aqueous phase. The total volumetric ratio of the organic phase to the aqueous phase was adjusted to 1 : 9. The liquid mixture was homogenized by ultrasonication, resulting in a stable oil-in-water (O/W) emulsion. The emulsion was stable for days at ambient and static conditions. The hydrodynamic diameter of the emulsion containing MNPs was around 187.9 ± 2.1 nm with a polydispersity index (PDI) of 0.248, as measured by dynamic light scattering (DLS). The emulsion was heated to 45 °C for 30 min to evaporate the chloroform, during which the color changed from turbid brown to translucent dark yellow. The structure and morphology of the MLs were observed by TEM (Fig. 1D). The efficiency of MNP insertion into the bilayers of the liposomes was significantly improved. DLS analysis revealed that the size and PDI of the MLs were 173.9 ± 1.8 nm and 0.292, respectively, very similar to size of the emulsion. These results indicate that the MLs originated from the O/W emulsions, and chloroform guided the MNPs into the bilayer membrane and significantly increased the insertion efficiency. We believe the main principle behind the enhanced insertion efficiency is that the MNPs are more familiar with the ‘phospholipid–chloroform mixed phase’ than phospholipids alone in an aqueous phase. Chloroform presumably functioned as a mediator between the hydrophobic surfaces of the nanoparticles and the hydrophobic parts of phospholipids. Finally, when chloroform was removed by evaporation, the MNPs were still able to interact with the hydrophobic alkyl chain of the phospholipids, leading to a high insertion efficiency into the bilayer (Fig. 1C). When the phospholipids and the MNPs were combined in the aqueous phase without chloroform, the phospholipids readily assembled into a lamellar structure, and MNP insertion was not possible owing to their large size.

**Fig. 2:**
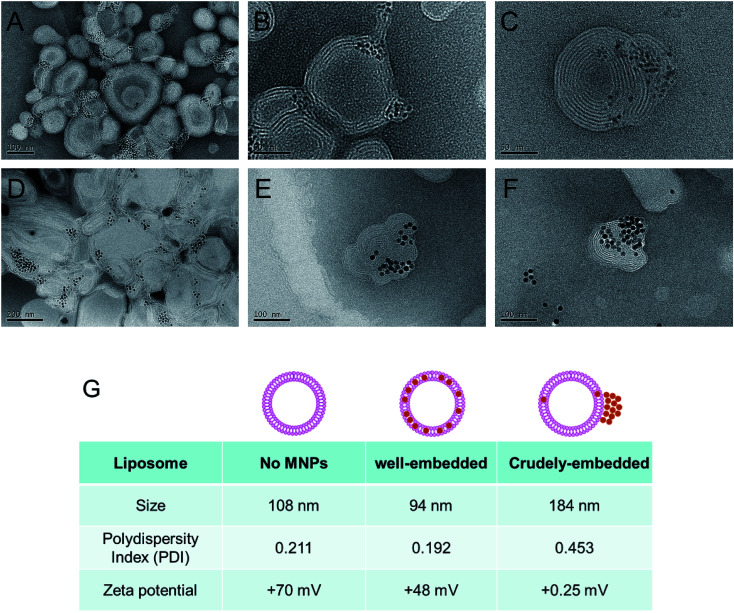
Preparation of MLs by sonication and chloroform-assisted methods. (A–C) TEM images of MLs with 6 nm MNPs. (D–F) The MLs with 15 nm MNPs. The chloroform was evaporated under sonication. (G) Size and zeta potential of plain DOPC/DOTAP liposomes, MNP well-embedded MLs and crudely-embedded/non-embedded MLs with 6 nm MNPs.

We found that the evaporation of chloroform from the O/W emulsion in stagnant states gives rise to a relatively wide size distribution of MLs. Therefore, to prepare more uniform MLs, we decided to evaporate chloroform in more vigorous conditions. The O/W emulsion composed of DOPC, DOTAP, and MNPs was prepared by the same method, and chloroform was then evaporated by increasing the temperature to 45 °C under ultrasonication. This process resulted in smaller (97.2 ± 0.7 nm in diameter) and more uniformly (0.192 PDI) sized MLs (Fig. 2G). Moreover, no precipitation or aggregation of the MNPs was observed in the ML suspension. TEM images of the MLs showed smaller, individual MLs (Fig. 2A–C). To further optimize the ML composition, we adjusted the weight ratios between the total amount of phospholipids and the MNPs; the optimal weight ratio for insertion was 20 : 1. We also tried to optimize the phospholipid ratio of DOPC to DOTAP from 6 : 4 to 9 : 1, but no significant differences were observed. Nevertheless, our results provide compelling evidence that chloroform guides the insertion of large inorganic nanoparticles into the narrow space of the phospholipid membrane composed of DOPC/DOTAP.

### 3.3. Preparation of MLs with 15 nm MNPs by solvent-guided methods

To explore the application of the chloroform guiding method to even larger nanoparticles, we evaluated the insertion of ∼15 nm MNPs into the phospholipid bilayers composed of DOPC/DOTAP. In this case, the MNPs were inserted into the phospholipid bilayers (Fig. 2D–F). Even though chloroform was previously employed for similar purposes, our findings indicated that larger nanoparticles (6 nm and 15 nm) could be inserted into the membrane more efficiently than those evaluated previously (4.1 nm).^22^ To explore the guiding effect of chloroform on other phospholipid formulations, we tested l-α-phosphatidylcholine (from egg yolk; egg PC) liposomes. The thickness of each phospholipid membrane is believed to be similar. As shown in Fig. S1,† both 6 nm and 15 nm MNPs could be inserted into the egg PC liposome.

### 3.4. HER2-targeted isolation of cancer cells using MLs

**Fig. 3:**
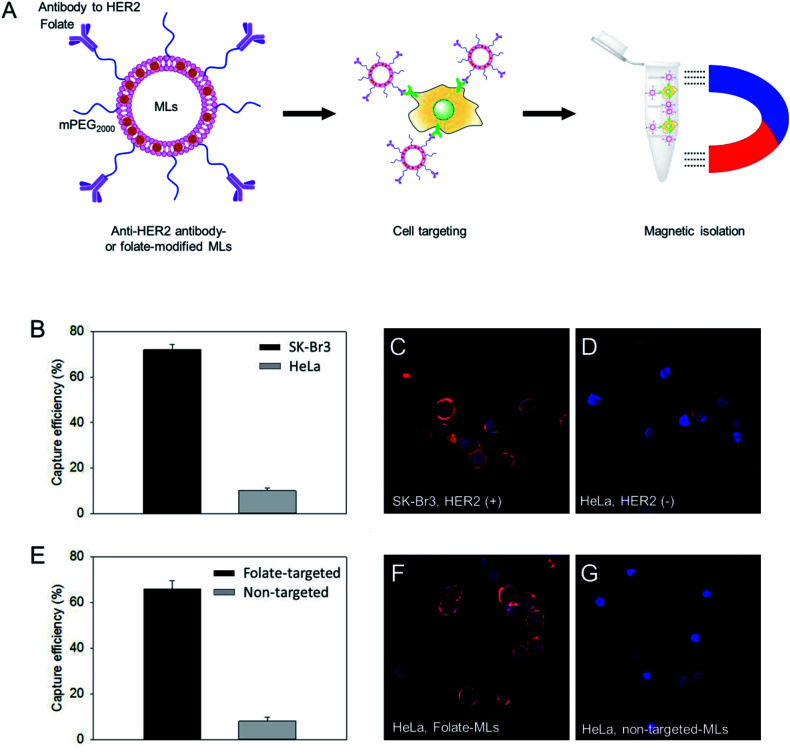
Cancer cell isolation test. (A) Designing cell targetable MLs binding to HER2 or folate receptor, and magnetic isolation using them. (B) Isolation efficiency of anti-HER2 antibody-modified MLs. SK-Br3 is a HER2 positive cell, and HeLa is a HER2 negative cell. Binding of the anti-HER2@MLs (C) to SK-Br3 or HeLa (D) were observed by confocal microscope. (E) Isolation efficiency of folate-modified MLs and non-targeted MLs. HeLa was used as a model cell. Binding of the folate@MLs (F) or non-targeted MLs (G) to HeLa.

DOPC/DOTAP (7/3 molar ratio) with 6 nm MNPs was used for the preparation of cell targeting MLs based on its higher embedding efficiency and yield than those for 15 nm MNPs (Fig. S3†). The surface of the ML was PEGylated using DSPE-mPEG_2000_ and NHS-PEG_3400_-maleimide (95 : 5 molar ratio). Then, the MLs were modified with thiolated antibodies against human epidermal receptor 2 (HER2), which is a specific membrane marker for breast cancer cells, through the maleimide/thiol reaction.^32^ Using the HER2 antibody-functionalized MLs, we tested the isolation efficiency of SK-Br3, a HER2-positive cancer cell, and compared it with HER2-negative HeLa cancer cells (Fig. 3A). The MLs were incubated with the cancer cell suspension (5 × 10^4^ cells per mL) in serum-free media. After 15 min, a neodymium magnet was used to retrieve the MLs along with the associated cells. After washing, cells were counted to determine the cell isolation efficiency. A 75% isolation efficiency was obtained for SK-Br3, mostly due to the binding of antibody-targeted MLs to the HER2 receptor on the cell surface (Fig. 3B). In contrast, when the MLs were used against HER2-negative HeLa cells, only 9% of cells could be retrieved. To further analyze the binding of MLs to cancer cells, fluorescent dye-labeled oligonucleotides were loaded onto the MLs *via* electrostatic interactions. Confocal microscopy clearly showed that antibody-modified MLs could bind to the HER2-positive SK-Br3 cell surface with high efficiency (Fig. 3C), whereas very low binding of MLs was observed on HeLa cells, presumably due to nonspecific interactions (Fig. 3D).

### 3.5. Folate receptor-targeted isolation of cancer cells using MLs

Similar to antibody-functionalized MLs, we prepared folate-modified MLs. The folate receptor is overexpressed in many cancer cells, and folic acid-functionalized liposomes and nanoparticles are widely used for targeted drug delivery to tumors.^33,34^ The surface of MLs prepared with the same lipid composition and ∼6 nm MNPs were PEGylated and functionalized with folic acid by treatment with a mixture of DSPE-PEG_2000_ and DSPF-PEG_2000_-folate (95 : 5 molar ratio). Folate receptor-expressing HeLa cells were used as a model to check the cell isolation efficiency. Compared to non-targeted PEGylated MLs, the folate-targeted MLs showed a significantly higher capacity to isolate HeLa cells (Fig. 3E). The folate-mediated targeting of MLs was also confirmed by confocal imaging. Contrary to only PEGylated MLs, HeLa cells treated with folate-modified MLs displayed much stronger fluorescence signals (Fig. 3F and G) on the cell surface. These results suggest that MLs with large (∼6 nm) MNPs could be used for the detection and analysis of circulating tumor cells.

### 3.6. Nuclear delivery of oligonucleotides using cationic MLs

**Fig. 4:**
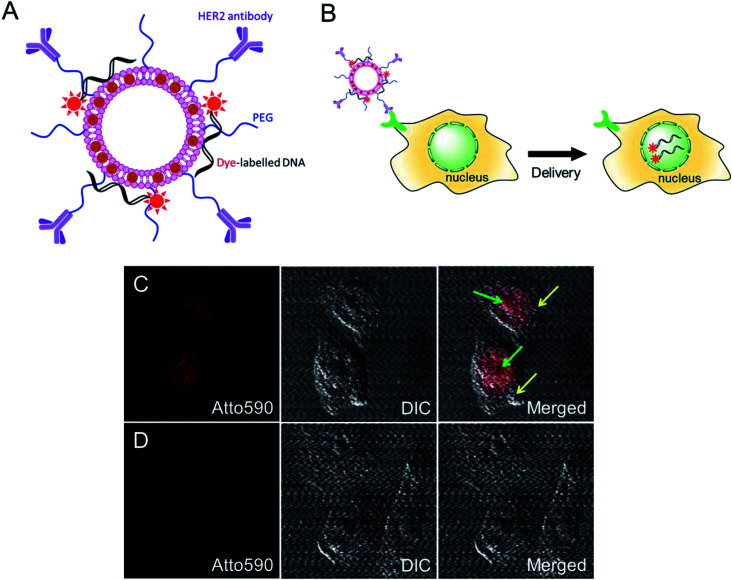
HER2 targeted intracellular delivery of oligo-DNA loaded MLs. (A) Schematic of designed MLs with loaded dye-labeled oligonucleotides (18 nt) and HER2-antibody. (B) Schematic of HER2-targeted delivery of oligonucleotides by MLs into the nucleus. (C) Delivery of atto590-labelled oligo DNA into the nucleus by anti-HER2 antibody-modified MLs. The fluorescence signal of oligo DNA could be discovered mainly in the nucleus of the cells. Green arrows indicate the nucleus and yellow arrows indicate cytoplasm. (D) No intracellular fluorescence signal in the cells treated with non-targeted PEGylated MLs.

We further investigated the intracellular delivery of oligonucleotides using our MLs as nano-carriers (Fig. 4A). The anti-HER2-conjugated MLs loaded with fluorescent-labelled oligonucleotides were efficiently delivered to the cancer cells and, interestingly, the fluorescence signals were predominantly detected inside the nucleus (Fig. 4B and C). Cationic liposomes facilitate endosomal escape and the translocation of oligonucleotides to the nucleus.^29,35^ A control study using DOPE/DOTAP cationic liposomes also showed the accumulation of oligonucleotides in the nucleus *via* non-specific uptake (data not shown). In contrast, PEGylated MLs were not taken up by cells, as evidenced by the lack of an intracellular fluorescence signal. The PEG coating endows MLs with stealth properties and reduces non-specific cell uptake (Fig. 4D).^36^ MLs are therefore capable of the intracellular delivery of loaded cargo and could be used for targeted drug or gene delivery applications.

## 4. Conclusions

We demonstrated the guiding effect of chloroform for the insertion of MNPs with average diameters of 6 nm and 15 nm into liposome phospholipid bilayers with a thickness of 3.4 nm. Unlike the conventional approach, which relies solely on spontaneous hydrophobic interactions, the addition of chloroform during the hydration of the lipid film enhanced the nanoparticle insertion efficiency. We successfully prepared the MLs with two different liposomes, with particularly focusing on the DOPC/DOTAP lipid formulation. By modifications with an anti-HER2 antibody and folate, the specific isolation of cancer cells was possible. These MLs have potential for magnetic-guided biomedical applications, *e.g.*, the isolation of circulating tumor cells, MRI contrast agents, hyperthermia therapy, and controlling cellular uptake by magnetic guidance.^37,38^ Toxicity of such types of liposomal materials has been investigated in terms of histological, hematological and genetical aspects for mice experiments. Limited toxicity was observed at low dose.^39^ In addition, the nanoparticle-inserted liposomes and their preparation method provide be a model system for cellular membranes with embedded channel/receptor molecules in the phospholipid bilayers.

## Conflicts of interest

There are no conflicts of interests to declare.

## Supplementary Material

RA-009-C9RA02529D-s001
