# Supplementary material for: Magnetoliposomes with size controllable insertion of magnetic nanoparticles for efficient targeting of cancer cells
Source: RSC Adv. 2019 May 14;9(26):15053–60. doi: 10.1039/c9ra02529d (PMC9064235; doi:10.1039/c9ra02529d)
Supplement: RA-009-C9RA02529D-s001 [file RA-009-C9RA02529D-s001.pdf]

Electronic Supplementary Material (ESI) for RSC Advances.
This journal is © The Royal Society of Chemistry 2019

Supplementary information

Magnetoliposomes with size controllable insertion of magnetic nanoparticles for efficient targeting of cancer cells

Won Il Choi[1], Abhishek Sahu[2], Frederik R. Wurm[3] and Seong-Min Jo[3,*]

[1]*Center for Convergence Bioceramic Materials, Convergence R&D Division, Korea Institute of Ceramic Engineering and Technology, 202, Osongsaengmyeong 1-ro, Osong-eup, Heungdeok-gu, Cheongju, Chungbuk 28160, Republic of Korea.*

[2]*School of Materials Science and Engineering, Gwangju Institute of Science and Technology, 123 Cheomdan Gwagi-ro, Oryong dong, Buk-gu, Gwangju 61005, Republic of Korea.*

[3]*Max Planck Institute for Polymer Research, Ackermannweg 10, D-55128 Mainz, Germany*

Correspondence to Seong-Min Jo

: jos@mpip-mainz.mpg.de, seongmini@gmail.com

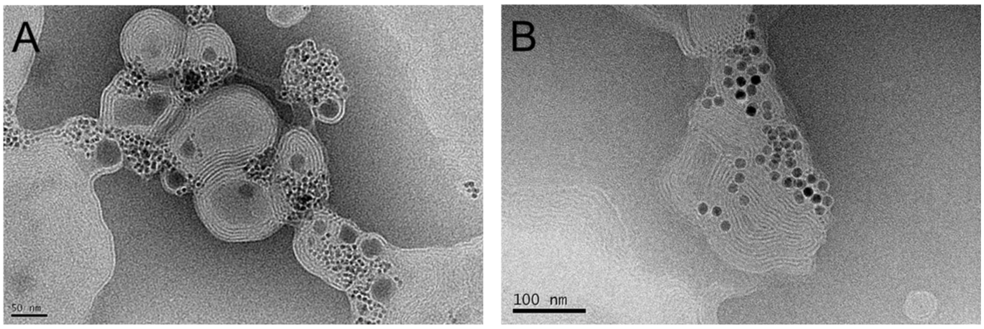

Figure S1. Insertion of 6 nm MNPs **(A)** and 15 nm MNPs **(B)** to egg PC liposomes

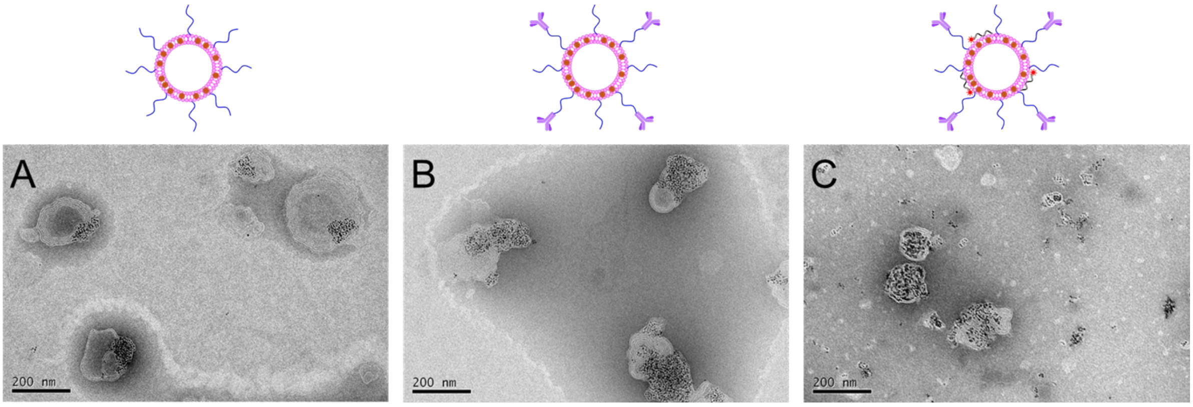

Figure S2. MLs after modification with NHS-PEG3400-Maleimide **(A)**, anti-HER2 antibody **(B)** and oligo-DNA **(C)**.

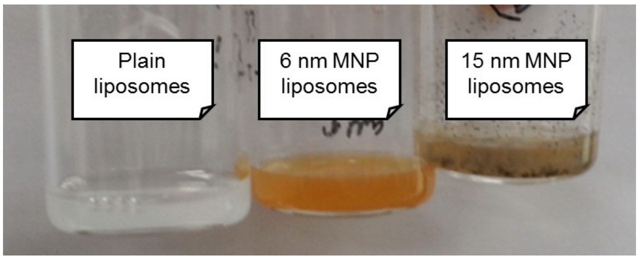

Figure S3. Liposomal suspension of plain DOPC/DOTAP (7/3) (left), with 6 nm MNPs (center) and with 15 nm MNPs.
